# Ethical climate in healthcare: A systematic review and meta-analysis

**DOI:** 10.1177/09697330231177419

**Published:** 2023-07-17

**Authors:** Ryan Essex, Trevor Thompson, Thomas Rhys Evans, Vanessa Fortune, Erika Kalocsányiová, Denise Miller, Marianne Markowski, Helen Elliott

**Affiliations:** Institute for Lifecourse Development, 4918 University of Greenwich, London, UK

**Keywords:** Ethical climate, systematic review, meta-analysis

## Abstract

**Background:**

Ethical climate refers to the shared perception of ethical norms and sets the scope for what is ethical and acceptable behaviour within teams.

**Aim:**

This paper sought to explore perceptions of ethical climate amongst healthcare workers as measured by the Ethical Climate Questionnaire (ECQ), the Hospital Ethical Climate Survey (HECS) and the Ethics Environment Questionnaire (EEQ).

**Methods:**

A systematic review and meta-analysis was utilised. PSYCINFO, CINAHL, WEB OF SCIENCE, MEDLINE and EMBASE were searched, and papers were included if they sampled healthcare workers and used the ECQ, HECS or EEQ.

**Ethical consideration:**

Ethical approval was not required.

**Results:**

The search returned 1020 results. After screening, 61 papers were included (*n* = 43 HECS, *n* = 15 ECQ, *n* = 3 EEQ). The overall sample size was over 17,000. The pooled mean score for the HECS was 3.60. Mean scores of individual studies ranged from 2.97 to 4.5. For the HECS studies, meta-regression was carried out. No relationship was found between the country of the studies, the study setting (ICU v non-ICU settings) or the mean years of experience that the sample had. For the ECQ, sub-scales had mean scores ranging from 3.41 (instrumental) to 4.34 (law) and were all observed to have significant and substantial heterogeneity. Three studies utilised the EEQ so further analysis was not carried out.

**Conclusions:**

The above results provide insight into the variability of scores as measured by the HECS, ECQ and EEQ. To some extent, this variability is not surprising with studies carried out across 21 countries and in a range of healthcare systems. Results also suggest that it may be that more local and context specific factors are more important when it comes to predicting ethical climate.

## Introduction

There is a rapidly growing body of research exploring ethical climate in healthcare settings. While the vast majority of this work can be found in the nursing literature, there is an increasing recognition of the importance of this concept amongst other health professions and since the declaration of the COVID-19 pandemic. This is perhaps unsurprising as ethical climate has been found to be related to both health worker and patient wellbeing, impacting the delivery of care and patient safety. In this study, we sought to analyse the literature that has explored ethical climate, examining perceptions of ethical climate amongst health workers and whether any differences in perceptions of ethical climate could be attributed to study or sample characteristics.

## Background

Ethical climate refers to the shared perception of ethical norms and sets the scope for what are ethical and acceptable behaviours within teams, groups and organisations.^1^ It is distinct from other moral concepts, as it has a primary focus on ‘social context in organizations [and how this] influences ethical behaviour of employees through fostering their collective moral reasoning’.^2^ Drawing on a several concepts in moral philosophy, ethical climate was introduced by Victor and Cullen^1^ and has since been utilised in a growing body of empirical work, including in research in healthcare settings. Amongst this work ethical climate has been found to influence job satisfaction, perceived workplace support and commitment^3^ and intent to leave a position.^4^ In addition to having an impact on staff wellbeing, ethical climate has also been linked to the delivery of health services and patient safety, with more negative ethical climate linked with poorer service delivery, including competence in relation to clinical and ethical issues.^5^ In one study, nurses who described their ethical climate to be more positive were less likely to make medical errors than those who appraised their ethical climate more negatively.^6^ Ethical climate has also been found to be related to moral distress.^7^ While moral distress has been the subject of conceptual debate, it generally refers to the unease felt by an individual where their ability to carry out an ethical action has been restricted in some way. Beyond having important impacts on healthcare staff and the delivery of health services, ethical climate itself is influenced by a complex range of factors, including organisation culture, leadership, policies, procedures and team structure, to name a few.^8^ Ethical climate is also not static, as organisations and teams are dynamic, ethical climate shifts across time and place and varies within teams and organisations.^2^

While the health workforce was already facing several pressing challenges, the COVID-19 pandemic has made research in this area even more pressing, with the pandemic placing increased pressure on the delivery of health services worldwide. These issues are also gaining increasing attention from major professional bodies. In the UK ,for example, the British Medical Association recently commissioned a survey to explore moral distress amongst its members.^9^ Emerging evidence suggests that ethical climate can have an important role in buffering potential stressors exacerbated by the pandemic.^10^

The majority of the empirical research which has explored ethical climate has utilised three instruments.^8^ The first instrument, the Ethical Climate Questionnaire (ECQ) was developed by Victor and Cullen^1^ and focuses on organisational ethical climate more generally. The ECQ contains 26 items that investigate 5 types of ethical climates, namely, caring, rule, instrumental, professionalism and independence. The Hospital Ethical Climate Survey (HECS) was developed by Olson^11^ and was originally designed to be used with nurses. The scale also has 26 items organised according to the relationships of peers, patients, managers, the hospital and physicians. The HECS has also been shortened and adapted for use with other healthcare workers more generally.^12^ The Ethics Environment Questionnaire (EEQ) was developed by McDaniel.^13^ It has 20 items and like the HECS was designed to be used in healthcare settings. For each of these scales, a higher score represents a favourable or more positive ethical climate. Each of these scales has been validated and shown to have good psychometric properties. A recent scoping review found that amongst the quantitative studies exploring ethical climate in healthcare settings, 22 utilised the HECS, 16 used the ECQ, while 5 used the EEQ,^8^ as these instruments are the most widely used we have focused on these below.

Given the increasing number of studies exploring ethical climate and the vastly different nature of these, the objectives of this study were to analyse and 1) explore perceptions of ethical climate as measured by the Ethical Climate Questionnaire (ECQ), the Hospital Ethical Climate Survey (HECS) and the Ethics Environment Questionnaire (EEQ) and to 2) examine whether ethical climate is related to study or sample characteristics, for example, the profession in question, the country the data was collected in, and whether the data was collected pre or during the COVID-19 pandemic.

## Methods

A systematic review was carried out to identify all relevant studies examining ethical climate amongst health workers. PRISMA guidance was followed^14^ and a study protocol was registered with Open Science Framework (https://doi.org/10.17605/OSF.IO/8S4H6).

### Search strategy

A search was carried out on 01/09/2022, utilising the following databases: PSYCINFO, CINAHL, WEB OF SCIENCE, MEDLINE and EMBASE. The final search terms were: (‘ethical climate’ OR ‘ethical environment’) AND (doctor OR physician OR clinician OR ‘medical practitioner’ OR nurs* OR ‘health profession*’ OR healthcare OR ‘health care’ OR pharmac* OR dentist OR midwi* OR dieti* OR therap* OR paramed* OR physiotherap * OR radiograph* OR Radiolog* OR surg* OR psycholog* OR ‘health worker’ OR hospital OR paramedic OR ambula* OR Carer OR ‘operating department practitioner’ OR ‘art therap*’ OR ‘biomedical scien*’ OR chiro OR podiatry* OR ‘clinical scien*’ OR dietician OR ‘occupational therap*’ OR orthoptists OR ‘speech and language’ OR ‘physical therap*’). We carried out a further manual search of references lists to identify further studies that were eligible.

### Eligibility criteria

No time or language limits were set. Studies were included if they reported on a sample of healthcare workers. We defined healthcare workers as ‘a person associated with either a specialty or a discipline and who is qualified and allowed by regulatory bodies to provide a healthcare service to a patient’.^15^ Healthcare professionals therefore included doctors, nurses, physiotherapists, dieticians and paramedics, among others. In line with this definition, we did not include studies that had a sample of staff that were unregulated (i.e. hospital cleaners or porters, for example).

Studies also had to report on ethical climate as measured by either the Hospital Ethical Climate Survey (HECS),^16^ the Ethical Climate Questionnaire (ECQ)^1^ or the Ethics Environment Questionnaire (EEQ).^13^ Variations of these scales were included where scores could easily be transformed (i.e. if scored on a different scale) and where the scale was validated (i.e. translations of the scale).

### Screening and data extraction

Screening was undertaken in two phases. A first screen was carried out independently by TE and DM examining the tile and abstract of articles. A second, full text screen was then carried out by TE and DM. Disagreements were resolved through discussion with the team and RE. All authors contributed to data extraction, with data extracted independently by at least two authors for each study. HECS, ECQ and EEQ scores, along with details about the study country, sample details and sample size, whether the study was carried out during the COVID-19 pandemic, along with the mean age and experience of the sample were extracted.

While we had planned to extract correlation coefficients for related scales, we only found a small number of studies that had extractable data. The most commonly measured outcomes were job satisfaction (5 studies, using 3 different scales), organisational commitment (3 studies using 2 different scales) and moral distress (10 studies, using 3 different scales). These scales were correlated across the three scales of interest in this study (HECS, ECQ and EEQ). Given the low number of studies we opted not to conduct any further analysis.

### Quality appraisal

All studies included were cross sectional, so the Appraisal tool for Cross-Sectional Studies (AXIS) was utilised.^17^ Each paper was independently assessed and scored on this scale.

### Data transformation and analysis

While a number of studies were excluded because scales were modified or no data could be extracted, where possible data was transformed. This involved transforming total scores to mean scores. It also involved transforming scales, that is, a number of studies had scored ECQ (scored on a 1–6 Likert scale) and HECS (scored on a 1–5 Likert scale) on different scales. In this case, scales were either divided by the number of response options and multiplied by the recommended number of response options. For example, for a study that scored the HECS on a 1–4 scales, scores were divided by 4 and multiplied by 5.

Meta-analysis was used to systematically synthesise the findings of the studies retrieved from the search. Mean scores and standard deviations were pooled using a random effects model with tests for heterogeneity. A meta-regression was carried out to explore if study characteristics impacted scores. The metafor package in R^18^ was used to carry out this analysis.

### Heterogeneity

The existence of heterogeneity was explored with Cochran’s Q statistic (where *p* < 0.05 indicates heterogeneity is present). The magnitude of the variation in effect sizes across studies with Higgin’s *I*^2^ statistic was also utilised. This statistic estimates the proportion of variance in effect sizes due to true heterogeneity (from 0% to 100%), with higher values representing greater inconsistency in effect size across studies. Finally, we also report τ as a measure of heterogeneity for each comparison, which gives the SD of the effect size estimates.

## Results

**Figure 1.**
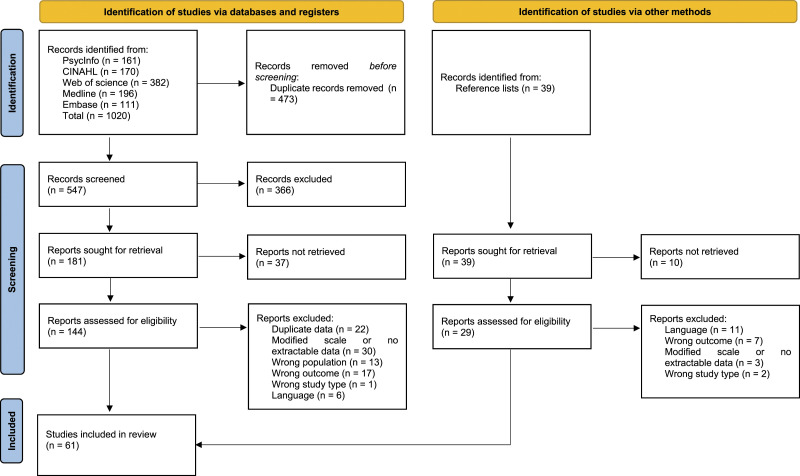
PRISMA 2020 flow diagram.

The search returned 1020 results. Results were imported into Rayyan^19^ where duplicates were removed, leaving 547 papers. A title and abstract screen left 181 articles, a full text screen was then carried out. A further 39 papers that were found in reference lists of included papers were also screened. After screening, 61 papers met the inclusion criteria; 43 that reported HECS scores, 15 that reported ECQ scores and 3 that reported EEQ scores. Amongst all of the studies, only eight studies had participants that were from interdisciplinary or allied health backgrounds; the remaining studies sampled nurses. The search results are summarised in Figure 1. After addressing risk of bias and study quality, below we will address each of our research questions, namely, 1) perceptions of ethical climate and 2) whether ethical climate is related to study or sample characteristics as measured by the HECS, ECQ and EEQ.

### Risk of bias and study quality

The overall quality of the studies included in this review was good. That is, most studies met most of the criteria laid out in the AXIS. To provide an overview of the quality of studies, a score out of 20 was calculated, counting the number of times a paper had met each criteria. For two criteria in relation to response rate and potential response bias and conflicts of interest, we counted ‘no’ responses. The mean score for all studies was 15, with studies ranging from 10 to 18, meaning all studies met at least half of the AXIS criteria. Few studies considered explicit justification of sample size and addressing and reporting non-response. A summary of these results is included in supplementary file A.

### Hospital ethical climate

**Figure 2.**
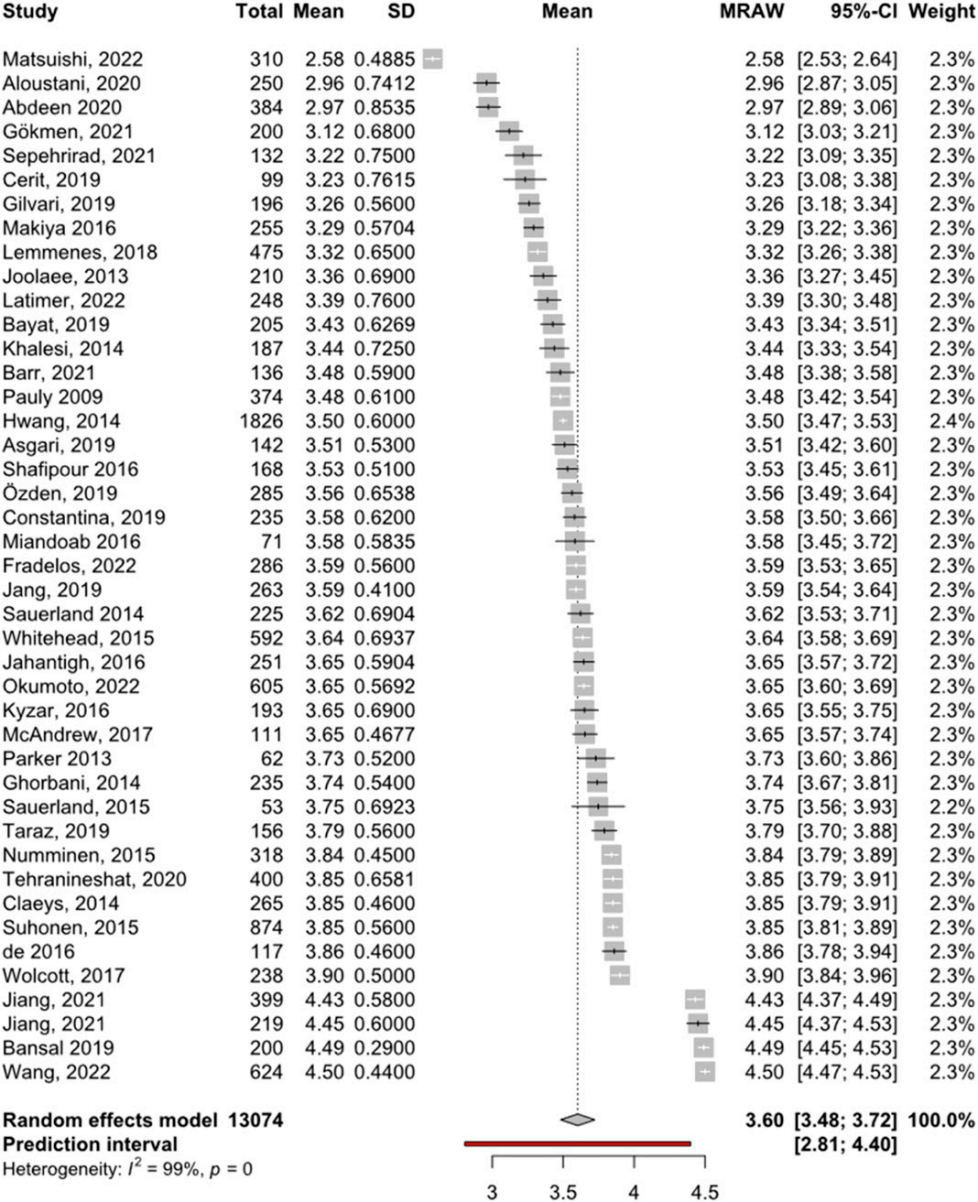
Forest plot of means and standard deviations of total HECS scores.

Studies that utilised the HECS (*n* = 43) were geographically diverse. The majority of studies were carried out in Iran (*n* = 13) 2^20,21,22,23,24,25,26,27,28,29,30,31,32^ and the US (*n* = 10).^33–42^ Several studies were carried out in Asia (*n* = 11)^10,43–51^ and Europe (*n* = 6).^52–57^ One study was carried out in Australia,^4^ Canada^58^ and Egypt,^7^ respectively. Studies had a pooled sample size of 13,074, with all studies sampling nurses, except three^28,37,54^ which had interdisciplinary samples. The pooled mean score for the HECS was 3.60 (95% confidence interval 3.48–3.72). Mean scores of individual studies ranged from 2.97 to 4.5. Significant (Q = 8051.48, *p* < 0.001) and substantial heterogeneity (*I*^2^ = 99.5%, τ^2^ = 0.15) were observed, meaning study means and standard deviations varied significantly between the studies. Results are summarised in Figure 2.

To explore this heterogeneity and if any other factors explained this variation, a meta-regression was carried out. Three variables were included, country of study (high income and low and middle income countries), setting of the study (intensive care/neonatal intensive care or other settings) and mean years of experience as a health worker. While we had planned to run further analyses, this was not possible; for example, there were only three studies that examined ethical climate during the COVID-19 pandemic. Interprofessional comparisons were also not possible, owing to almost all studies sampling nurses. Results indicated that scores were not impacted by setting (*p* = 0.70), country (*p* = 0.89) or years of experience (*p* = 0.29). These results are summarised in Table 1.

**Table 1.** Meta-regression results.

Variable	Number of studies	Coeff	S.E.	*p*	95% CI
Setting	18	0.14	0.36	0.70	−0.63–0.91
Country	18	0.03	0.23	0.89	−0.45–0.52
Experience	18	−0.03	0.03	0.29	−0.08–0.03

A sensitivity analysis was conducted with outliers removed,^10,46,47,50,51^ this exclusion however had little impact on the overall results, so we opted to retain these studies.

### Ethical climate questionnaire

Studies that utilised the ECQ (*n* = 15) were as geographically dispersed as those utilising the HECS. The majority of studies were carried out in the US (*n* = 4),^59–62^ China (*n* = 2)^63,64^ and Egypt (*n* = 2).^3,65^ The remainder of the studies were carried out in Bosnia and Herzegovina,^66^ Cyprus,^67^ Ethiopia,^68^ Iran,^69^ Israel,^70^ Taiwan^71^ and Turkey.^72^ ECQ studies had a pooled sample size of 4,442, with all studies sampling nurses, except two^62,63^ which had interdisciplinary samples. As the ECQ reports five ethical climate types, it was rarely reported as a total score; below we report the results for each sub-scale separately (note – not every study reported all sub-scale scores). Sub-scales had mean scores ranging from 3.41 (instrumental) to 4.34 (law) and were all observed to have significant and substantial heterogeneity. These results are summarised in Table 2 and forest plots for each sub-scale are included in supplementary file A.

**Table 2.** Meta-analysis of ECQ sub-scales.

ECQ sub-scale	Number of studies	Mean	95% CI	Range	Q (*p*)	*I*^2^, %	τ^2^
Care	15	3.71	3.29–4.14	2.36–5.26	2996.82 (<0.001)	99.5	0.59
Instrumental	12	3.41	3.03–3.79	2.48–4.72	1667.94 (<0.001)	99.3	0.36
Law	13	4.34	3.91–4.77	3.33–5.82	2266.10 (<0.001)	99.5	0.51
Rule	13	4.13	3.63–4.63	2.94–5.61	2276.89 (<0.001)	99.5	0.68
Independent	13	3.48	3.09–3.87	2.14–4.69	1011.96 (<0.001)	98.8	0.41

The feasibility of running further analyses was explored; however, we opted not to do so as there were not enough studies, with any further analysis likely to be underpowered.

#### Sensitivity analysis

A sensitivity analysis was conducted for each scale with outliers removed.^59,61,65,68,70^ This exclusion however had little impact on the overall results, so we opted to retain these studies.

### Ethical environment questionnaire

There were only three studies that utilised the EEQ and met our inclusion criteria.^73–75^ These studies were from Australia and the US, sampling nurses, audiologists and physiotherapists, respectively. Mean EEQ scores ranged from 3.23^75^ to 3.8.^73^ Because of the small number of studies, further analyses were not carried out.

## Discussion

This study gives insight into the nature and variability of ethical climate in healthcare, as measured by the HECS, ECQ and EEQ. The reviewed studies represent a sample of over 17,000 healthcare workers, the vast majority of which were nurses. HECS mean scores ranged from 2.97 to 4.5, with a mean score of 3.6 (95% CI 3.48–3.72). ECQ scores ranged from 3.41 for the care sub-scale to 4.34 for the law sub-scale. HECS scores were not related to the country or setting of the research or the years of experience of the sample. For both the HECS and ECQ, scores were observed to be highly heterogeneous, that is, mean scores varied significantly across studies. In some ways, this is not surprising given the nature of these studies, and the fact that they were carried out across 21 countries, within a range of healthcare systems and in a range of different teams. As we will discuss below, our analysis did not find any study and sample-related characteristics that explained this heterogeneity. In saying this however, within these results, there are a number of studies that could be considered outliers, with scores that were comparably higher or lower than most studies; in this respect these scores may be useful to inform future research in what may be considered an unusually high or low ethical climate score.

Beyond the range and nature of ethical climate scores as they related to these scales, these results also suggest that it may be that more local and context specific factors are more important when it comes to predicting ethical climate. That is, while we were limited in the data we could extract and analyse, caution should be exercised in making generalisations about factors such as country (i.e. low or high income countries) or the specific study setting (i.e. ICU compared to other settings), as the results here do not suggest these factors influenced ethical climate and any significant or predictable way. These findings are consistent with the broader literature. Although there has been limited research exploring the antecedents of ethical climate, studies that come from outside of healthcare settings also suggest that it may be organisations factors that are more influential, with studies focussing on ‘leadership and managerial practices, organizational practices, organizational and cultural context, and individual differences’.^2^

### Limitations

There are several limitations that are worth noting in regards to this review. First, there were multiple papers where data was incomplete or reported inconsistently. This was particularly the case with papers that utilised the ECQ, with the majority of papers either making a number of changes to how the scale was administered or scored. Where possible, we converted these scores, however, a number of papers were excluded because of this. Future research should be mindful of how scales were developed and intended to be administered. Second, there are several further limitations in relation to our analyses. Because of the limited number of studies and the nature of this data, we opted to fold our data with fewer categories (for example, with country and setting); this limits the conclusions that can be drawn in regards to differences between countries and different healthcare settings. We also could not calculate correlation coefficients between ethical climate and other scales for these reasons. While we extracted data related to years of experience of samples, this was not widely reported. Greater reporting of sample demographic characteristics, amongst other study characteristics, are recommended to maximise contributions to future meta-analyses. In addition to this, the vast majority of studies included in this review sampled nurses, this means that caution should be exercised in generalising these results to other healthcare workers. Future studies may also want to explore ethical climate scores alongside other variables, such as whether the study was carried out before or during the COVID-19 pandemic, which unfortunately was not possible in this paper.

## Conclusions

This study found ethical climate scores to be highly variable between studies and found no relationship between study characteristics and ethical climate scores. Analyses were limited by the data that could be extracted from studies. However, future studies should more comprehensively report the factors that are likely to impact ethical climate and any potentially confounding or context specific factors that may influence perceptions of ethical climate. In a practical sense, these findings re-enforce existing studies that suggest that ethical climate may be best addressed from the bottom-up in consultation with those delivering healthcare services by providing systems, policies and processes that facilitate ethical behaviour.
